# Individual Variations in Serum Melatonin Levels through Time: Implications for Epidemiologic Studies

**DOI:** 10.1371/journal.pone.0083208

**Published:** 2013-12-23

**Authors:** Leticia M. Nogueira, Joshua N. Sampson, Lisa W. Chu, Kai Yu, Gerald Andriole, Timothy Church, Frank Z. Stanczyk, Jill Koshiol, Ann W. Hsing

**Affiliations:** 1 Division of Cancer Epidemiology and Genetics, National Cancer Institute, Bethesda, Maryland, United States of America; 2 Cancer Prevention Institute of California, Fremont, California, United States of America; 3 Division of Urological Surgery, Washington University School of Medicine, St. Louis, Missouri, United States of America; 4 Department of Environmental Health Sciences, University of Minnesota School of Public Health, Minneapolis, Minnesota, United States of America; 5 Department of Obstetrics, Gynecology, and Preventive Medicine, Keck School of Medicine, University of Southern California, Los Angeles, California, United States of America; Universität Bochum, Germany

## Abstract

Melatonin, a marker for the circadian rhythm with serum levels peaking between 2AM and 5AM, is hypothesized to possess anti-cancer properties, making it a mechanistic candidate for the probable carcinogenic effect of circadian rhythm disruption. In order to weigh epidemiologic evidence on the association of melatonin with cancer, we must first understand the laboratory and biological sources of variability in melatonin levels measured in samples. Participants for this methodological study were men enrolled in the Prostate Lung Colorectal and Ovarian Cancer Screening Trial (PLCO). We measured serum melatonin levels over a five year period in 97 individuals to test if melatonin levels are steady over time. The Pearson correlation coefficient between two measures separated by 1 year was 0.87, while the correlation between two measures separated by 5 years was to 0.70. In an additional cross-sectional study of 292 individuals, we used Analysis of Variance to identify differences in melatonin levels between different lifestyle and environmental characteristics. Serum melatonin levels were slightly higher in samples collected from 130 individuals during the winter, (6.36±0.59 pg/ml) than in samples collected from 119 individuals during the summer (4.83±0.62 pg/ml). Serum melatonin levels were lowest in current smokers (3.02±1.25 pg/ml, p = 0.007) compared to never (6.66±0.66 pg/ml) and former (5.59±0.50 pg/ml) smokers whereas BMI did not significantly affect serum melatonin levels in this study. In conclusion, the high 5 year correlation of melatonin levels implies that single measurements may be used to detect population level associations between melatonin and risk of cancer. Furthermore, our results reiterate the need to record season of sample collection, and individual characteristics in order to maximize study power and prevent confounding.

## Introduction

Exposure to light at night has two major physiological actions: it 1) disrupts circadian rhythms and 2) suppresses the production of melatonin [1]. In 2007, the International Agency for Research on Cancer classified circadian rhythm disruption as a probable carcinogen to humans [2]. Circadian disruption, is mostly measured by shift work in epidemiologic studies, and is associated with several cancers [3]–[5] including prostate [6]–[11], breast [12]–[41], endometrium [16], ovarian [42], and colorectum [43], as well as non-Hodgkin lymphoma [44]. However, shift work studies lack detailed data for determining which aspects of circadian rhythm disruption, which includes melatonin levels, work and leisure activities, biological stress, ambient noise [45], food [46], [47], and chronotype [48], are associated with cancer risk. Incorporating biomarkers to epidemiologic studies can help not only in identifying the underlying mechanisms responsible for the circadian disruption-cancer association, but also in interpreting existing epidemiologic data [4].

Melatonin is excreted exclusively during the night by the pineal gland [49] and exposure to light at night interrupts melatonin secretion [50] and is associated with lower melatonin levels in observational studies [51]–[55]. Additionally, melatonin can reduce cancer cell proliferation and block cell invasion/metastasis [56], [57], providing biological plausibility to the role of melatonin in cancer. Furthermore, concentration of the major metabolite of melatonin, urinary 6-sulfatoxymelatonin (aMT6s), has been shown to be inversely correlated with breast cancer risk in most studies evaluating this association [58]–[62].

In addition to circadian rhythm, melatonin also displays seasonal rhythm. Melatonin levels have been shown to be higher during the winter in populations residing north of the 45^th^ parallel [51], [63]–[69], where differences in day length between seasons are more pronounced. However, the effect of seasons on melatonin levels in middle latitudes, where two thirds of the world population resides [70], is not clear. Finally, previous studies have shown that lifestyle-related cancer risk factors, such as body mass index (BMI) and smoking status, also affect urinary aMT6s levels in women [51], [62], [71]. However, the effects of these potential confounders have not been evaluated in serum melatonin levels or in men.

Serum is more commonly collected in epidemiological studies than morning urine and could be an important biologic resource for evaluating the role of melatonin on cancer. Morning urine aMT6s and serum melatonin levels are well-correlated [72] but represent different aspects of the melatonin profile [73]. While morning urine aMT6s is related to peak nocturnal melatonin levels [74], serum melatonin reflects the amount of melatonin circulating during the time of sample collection [73], [75]. Epidemiological studies often rely on a single serum sample commonly collected during daylight when melatonin levels are low [76]. Since the average serum melatonin levels over time are likely to be associated with disease, a non-representative single measurement will reduce the study's power to detect and quantify any tested association using melatonin as a biomarker [77]. Additionally, it is important to characterize the influence of possible cofounders on serum melatonin levels. Factors possibly influencing individual changes in melatonin levels over time that have been evaluated in epidemiologic studies conducted to date include subject's age and sample storage time. Melatonin levels decrease with age [51], [62], [71], [78], [79] and the correlation between repeated measurements of aMT6s decreases as time between measurements increases [51], [69], [71], [80]. However, the effect of age and sample storage time on serum melatonin levels has not yet been quantified.

While evaluating the association of melatonin with cancer, we need to appropriately collect, and ultimately weigh, observational evidence [81]. Being able to use the environmental and biological context to improve serum melatonin measurements accuracy is crucial for building observational evidence [82]. Hence, our objective was to determine the best approach for evaluating serum melatonin measurements in epidemiological studies. We measured individual serum melatonin variability over a 5 years period to determine representativeness of a single measurement, quantified the impact of time of the day during sample collection on study power to identify associations between serum melatonin levels and health outcomes, and evaluated potential confounders of these associations, such as smoking and BMI.

## Materials and Methods

### Study Population

The study population consisted of 300 non-Hispanic white men enrolled in the Prostate Lung Colorectal and Ovarian Cancer Screening Trial (PLCO), a randomized trial designed to determine the effectiveness of screening on cancers of the lung, prostate, ovary and colorectum mortality. Details on PLCO study, including Institutional Review Board approval, have been previously published [83]. In brief, men aged 55–74 years were randomly assigned to the screening or the control arm of the study at 10 screening centers between 1993 and 2001. The 300 individuals in this study had no prior history of cancers and, being in the screening trial arm of the study, had at least one prostate cancer screen before October 1^st^ 2003. Trial participants are volunteers recruited from the general population in Denver CO, Washington DC, Detroit MI, Pittsburg PA, Salt Lake City UT, Marshfield WI, and Minneapolis MI. We do not anticipate participation in the screening interferes with melatonin levels. Participants were those who completed a baseline questionnaire to elicit information on demographic characteristics and risk factors for cancer and returned at least one annual study update. This research was approved by the Department of Health and Human Services Institutional Review Board. Signed informed consent was required for eligibility.

**Figure 1:**
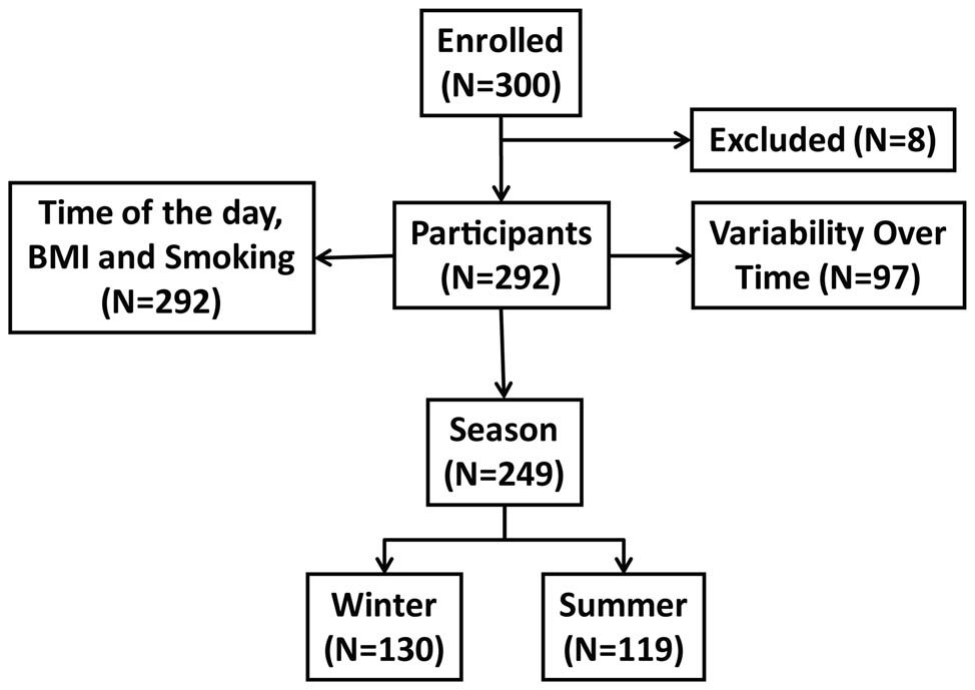
Study Design. From 300 subjects enrolled in the study, 292 were selected after quality control for being included in the study. All 292 were used in the evaluation of the effects of time of the day during sample collection, BMI, and smoking. For the evaluation of differences in melatonin levels according to season, 130 subject whose samples were collected during the winter and 119 subjects whose samples were collected during the summer were selected (Total = 249 subjects). Samples collected from 97 subjects in 4 different time points were used to evaluate changes in melatonin levels over time.

We used stored non-fasting serum that were processed and frozen within 2 hours of collection and stored at −70°C and excluded 3 samples that did not reach the minimum level of detection for the melatonin assay (0.5 pg/ml). In addition, we excluded 5 outliers based on the definition of the following characteristics: Cook's distance greater than 4/n (where n is number of observations), studentized residuals greater than 2, and leverage greater than (2k+2)/n (where k is the number of predictor variables and n is the number of observations). The final analytical dataset included 292 subjects (Figure 1). Inclusion of the outliers in the analysis did not change any of the reported associations but yield different estimates.

To evaluate the impact of season on serum melatonin level, we measured melatonin in baseline samples in 130 men whose blood was collected during the winter (December through March) and 119 (no overlap) men whose samples were collected during the summer (June through September). We focused on winter and summer since these are the seasons with the greatest difference in day length. All 292 subjects were included in the analysis to evaluate the effect of time of the day during blood collection, self-reported BMI, and smoking status (Figure 1).

To assess intra-person variation in melatonin levels over a 5-year period, we measured serum melatonin in 97 out of the 292 subjects. These 97 subjects gave blood at four time points, including baseline (T0), second (T2), fourth (T4) and fifth year (T5) of the PLCO follow up study (Figure 1). To ensure that we were evaluating individual rather than environmental variations, we selected subjects who had blood samples collected during the same season and at the same time of the day for all four time points for this evaluation. In addition, we limited the subject selection to those with all specimens collected between 7AM and 11AM, since melatonin levels are reported to be very low in the afternoon [76].

### Serum Melatonin Assay

Serum melatonin levels were measured using the Buhlmann RIA kit (ALPCO, Windham NH) as previously described [72]. Briefly, 0.5 ml of serum was used for the melatonin extraction. Melatonin was then reconstituted in assay buffer before being quantified by Radioimmunoassay (RIA) using antiserum with an iodinated tracer.

To evaluate technical variability, we included 2 replicate samples from 3 individuals in each batch. Also, to minimize the impact of assay/laboratory variation on the comparison of serum melatonin, at the lab, we paired the 130 samples collected during winter with the 119 samples collected during summer for the analysis. Each pair was placed next to each other within the same batch to minimize intra- and inter-assay variation.

In addition, for the 97 subjects with four samples from various time points, the samples from all four time points from the same subject were assayed within the same batch. All samples were identified by specimen ID only so that that the lab personnel were blinded to the status and identification of the sample.

### Statistical Analysis

#### Melatonin Variability over Time

To assess the correlation in melatonin levels over time (T0, T2, T4, T5), our primary objective, we measured the unadjusted Pearson correlation coefficient between each pair of time points (e.g. T0 vs. T2, T0 vs. T4, T0 vs. T5) among the 97 men with serial samples. We then evaluated all time points together to obtain a single estimate of the correlation, 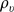 between the average melatonin levels for two consecutive years.

To define and estimate 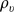 we assumed that the measured log-melatonin level, Y_it_, for individual i at time t is the individual's average log-melatonin level, M_it_, plus an error term, ε_it_, that accounts for unmeasured short term effects (e.g. jet lag, weather, lab error)
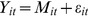
(1)We further assume that the individual's average log-melatonin level around time t can be described by the following function,
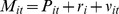
where P_it_ is ‘population’ average, r_i_ is a subject specific effect, and ν_it_ is the individual's deviation from their average. We further assume that ε_it_, r_i_ and ν_it_ are independent normally distributed random variables, 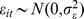, 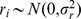 and 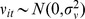, and that the individual's deviation from their own average is correlated over time, with the correlation between years t_1_ and t_2_ defined by 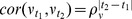.

We define the ‘population’ average to be the expected log-melatonin levels for samples that have been in storage for the same number of years and were collected from men of the same age. Let A_it_ and S_it_ denote age and years in storage respectively.
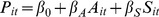
(2)The complete mixed model can be described by 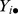 being normally distributed with mean 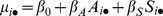 and variance
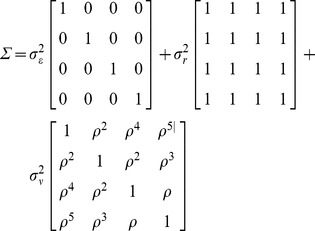
Additionally, we assessed the contribution of laboratory error to ε_it_. Here, we fit a mixed model with both the replicate quality control (QC) samples and baseline samples to estimate the intraclass correlation coefficient (ICC_rep_), or the correlation between replicate measures.

The effect of batch on log-melatonin levels was tested by ANOVA comparing models with and without batch. Mixed models allowed for within-subject correlation, and models were fit with only QC samples (p-value for batch = 0.51), only study specimen (p = 0.55), and both (p = 0.79). Batch did not have a significant effect. Thus, log-melatonin levels were not batch adjusted.

#### Environmental and Behavioral Factors Influencing Melatonin levels

To evaluate differences in melatonin levels between environmental and behavioral characteristics, our second objective, we used t-tests and χ^2^ to identify differences between the two season groups (winter N = 130 and summer N = 119) for continuous (age) and categorical (BMI, time of the day during blood collection, center location, and smoking) variables, respectively. Since there were no differences in age, BMI, time of the day during blood collection, and smoking between the two groups, we used Analysis of Variance (ANOVA) to evaluate differences in unadjusted log-melatonin levels between the winter and the summer.

To evaluate how baseline log-melatonin levels varied with respect to time of day during blood collection, BMI, and smoking status all 292 subjects were used. Time of day during blood collection was categorized into three blocks: 7AM–9AM, 10AM–12PM and 1PM–4PM. We used information on self-reported BMI and smoking status from the baseline questionnaire. BMI was categorized into normal (18–25 kg/m^2^), overweight (26–30 kg/m^2^), and obese (>30 km/m^2^), and smoking status was categorized into never smoker, current smoker, and former smoker. We used ANOVA to evaluate differences between each category. We also conducted a linear regression model in order to evaluate the relative impact of these variables on serum melatonin levels.

All data were analyzed using SAS 9.2 TS.

#### Estimates of power

In order to quantify how time of the day during sample collection affects study power to detect associations between melatonin and outcome of interest, our third objective, we assessed the cost or benefit of a non-standard study design with N subjects by calculating the number of subjects, N_eq_, which would provide equivalent statistical power for a study design that had one up-to-date measure per subject. We assumed the study goal was to detect an association, or correlation, that exists between an outcome and M_it_. Furthermore, we assumed analyses are stratified by A_it_ and S_it_, and conditional on M_it_, and that the outcome is independent of all previous melatonin levels (i.e. no lagged effect).

We first considered a study design that records *n* measurements within a “short” interval for each individual. Here, for time points t_1_ and t_2_ within a “short” interval we assumed 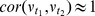 and 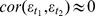. Then
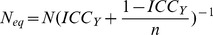
where 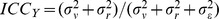. We then considered a study design that replaces a single up-to-date measure for time t_0_ with previously recorded measure from time t.
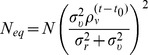



## Results

### Characteristics of the study population

For this study population mean age at baseline (standard deviation) was 62.88 (3.98) years. Additionally, the majority [N = 160 (54.98%)] of the participants were overweight, compared to 73 (25.09%) normal weight and 58 (19.93%) obese participants. Additionally, 168 (57.73%) of the participants were former smokers, 96 (23.99%) never smoked, and 27 (9.28%) were current smokers at baseline. A total of 158 (54.30%) samples were collected between 7AM–9AM, 83 (28.52%) were collected between 10AM–12PM, and 50 (17.18%) were collected between 1PM–4PM. Finally, 130 (44.67%) samples were collected during the winter, 32 (11.00%) were collected during the spring, 119 (40.89%) were collected during the summer, and 10 (3.44%) were collected during the fall (Table 1).

**Table 1:** Characteristics of the study population.

Characteristics	Mean (SD)
**Age**	62.88 (3.98) years
**BMI**	**N (%)**
**Normal**	73 (25.09)
**Overweight**	160 (54.98)
**Obese**	58 (19.93)
**Smoking**	
**Never**	96 (32.99)
**Current**	27 (9.28)
**Former**	168 (57.73)
**Time**	
**7AM–9AM**	158 (54.30)
**10AM–12PM**	83 (28.52)
**1PM–4PM**	50 (17.18)
**Season**	
**Winter**	130 (44.67)
**Spring**	32 (11.00)
**Summer**	119 (40.89)
**Fall**	10 (3.44)

### Melatonin Variability over Time

The variability of melatonin over time was assessed using 97 men aged 55 to 71 with samples collected at 4 time points (T0, T2, T4, and T5). The samples used in this analysis were in storage for between 4 and 15 years. The average correlation coefficient (95% Confidence Interval) for measurements separated by 1, 2, 4, and 5 years were 0.87 (0.81, 0.91), 0.80 (0.72, 0.86), 0.73 (0.63, 0.81), and 0.70 (0.59, 0.79) respectively, suggesting a reduction in correlation over time. Only a small proportion of the short-term variability is attributable to laboratory variability, 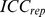 = 0.98 (0.005).

We estimated the short-term and long-term variability by fitting equations 1, 2, and 3 and found 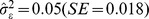, 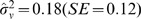, 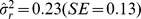 and 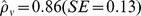. A small proportion of variability (11% = 0.05/0.46) can be attributed to short term changes in individual's melatonin level. These estimates suggest that the correlation between measurements taken at intervals of 1 year, 2 years, 5 years, and 10 years should be. 0.83, 0.78, 0.68, and 0.58.

Factors which could influence changes in melatonin levels during a 5 year period include, but are not limited to, age of the individual and sample storage time. For samples stored over the same time period, melatonin levels were expected to decrease by approximately 3% for each additional year of age (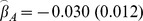, equation 2). For samples from individual(s) of the same age, melatonin levels were expected to decrease by 3.8% (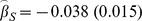) for each additional year of storage.

### Environmental and Behavioral Factors

**Figure 2:**
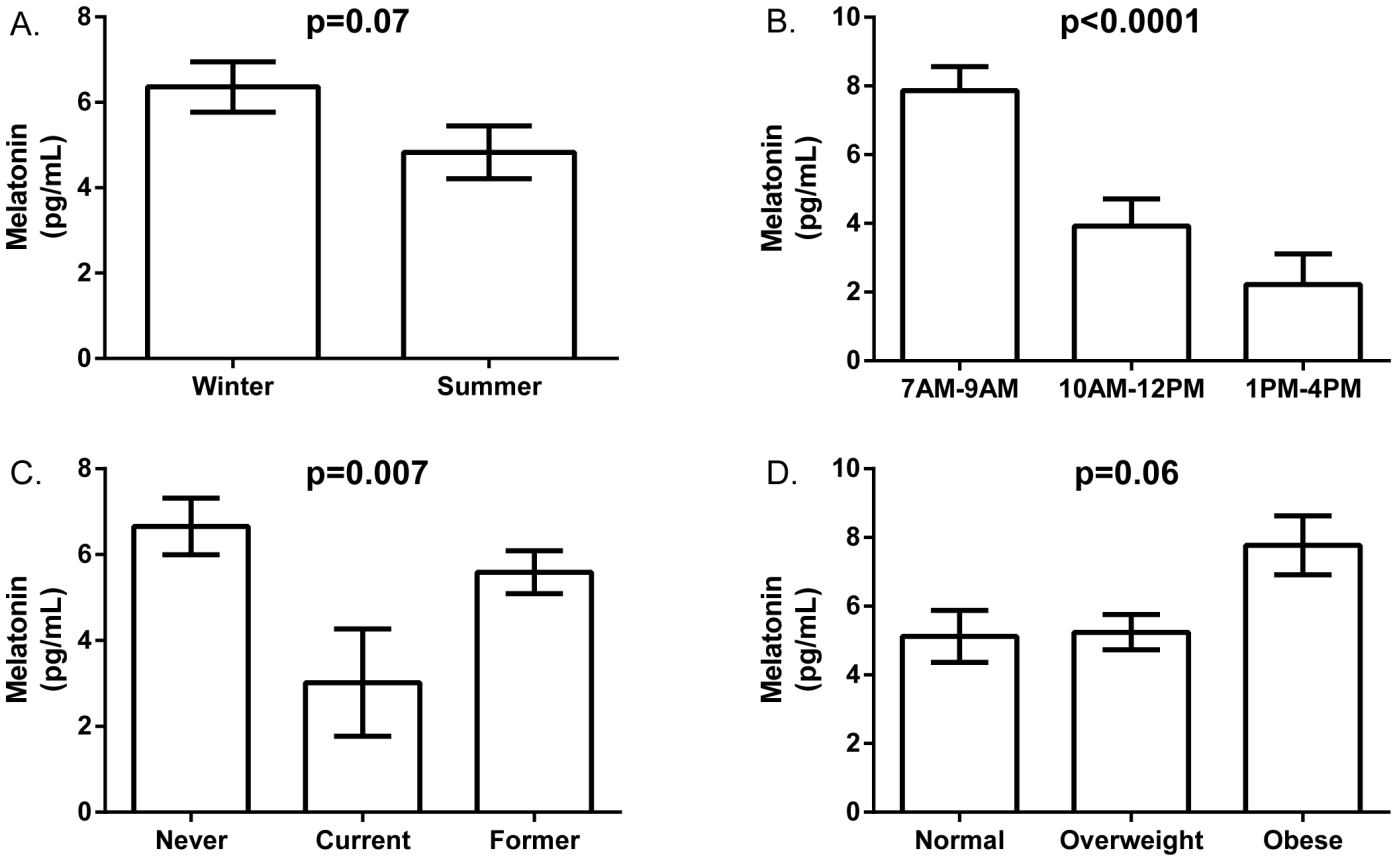
Sources of serum melatonin levels variability. **A**. Melatonin levels were higher during the winter [Mean = 6.36 (Standard Error of the Mean = 0.57) pg/ml, n = 130], although not statistically significantly different (P^MANOVA^ = 0.07) than summer values [4.83 (0.60) pg/ml, n = 119]. **B**. Mean serum melatonin levels were highest [7.86 (0.70 pg/ml), n = 158] in early morning 7AM–9AM, twice as high as serum melatonin levels collected between 10AM–12PM [3.92 (0.79) pg/ml, n = 83] and three times higher than samples collected between 1PM–4PM [2.22 (0.89) pg/ml, n = 50] (P^ANOVA^<0.001). **C**. Melatonin levels were lowest in current smokers [3.02 (1.25) pg/ml, n = 96, P^ANOVA^ = 0.007] compared to never smokers and former smokers [6.66 (0.66) pg/ml, n = 27, and 5.59 (0.50) pg/ml, n = 168, respectively]. **D**. BMI was not associated with melatonin levels (P^ANOVA^ = 0.059), being similar between normal weight [5.12 (0.76) pg/ml, n = 72], overweight [5.24 (0.51) pg/ml, n = 216], and obese [7.77 (0.86) pg/ml, n = 100] subjects.

Serum melatonin levels were slightly higher in the 119 samples collected during the winter [6.36 (0.59) pg/ml] compared to the 130 collected during the summer [4.83 (0.62) pg/ml], although this difference was not statistically significant (P^ANOVA^ = 0.07) **(** **Figure 2A** **)**. There were no differences in age, time of blood collection, BMI, or smoking history between the 130 and 119 subjects who had blood collected in winter or summer (Table S1).

The effects of time of day during blood collection, smoking, and BMI were assessed using the baseline measures in 292 men. As expected, serum log-melatonin levels decreased significantly with increasing time of day. Melatonin levels [mean (standard error of the mean)] were highest in serum collected in the early morning 7AM–9AM [7.86 (0.70) pg/ml, n = 158, P^ANOVA^<0.001], twice as high as serum collected in the 10AM–12PM [3.92 (0.79) pg/ml, n = 83) period, and three times higher than serum collected in the 1PM–4PM period [2.22 (0.89) pg/ml, n = 50] (**Figure 2B**).

In addition, mean (SEM) serum melatonin levels were lowest in current smokers [3.02 (1.25) pg/ml, n = 96, P^ANOVA^ = 0.007), compared to never smokers [6.66 (0.66) pg/ml, n = 27], and former smokers [5.59 (0.50) pg/ml, n = 168) **(** **Figure 2C** **)**. Serum melatonin levels were similar in normal weight and overweight subjects [5.12 (0.76) pg/ml, n = 72 and 5.24 (0.51) pg/ml, n = 216, respectively), and slightly higher in obese subjects [7.77 (0.86) pg/ml, n = 100, P^ANOVA^ = 0.06] **(** **Figure 2D** **)**.

Similarly, when evaluating the relative impact of these variables, time of the day during sample collection had the greatest effect on serum melatonin level (β = −4.00 and −5.44 for samples collected between 10AM–12PM and 1PM–4PM compared to 7AM–9AM). Smoking also had a significant relative effect (β = −3.16 for current smokers compared to never smokers). The effects of BMI and season on melatonin levels were not significant (Table 2).

**Table 2:** Relative impact of environmental and behavioral factors on serum melatonin levels.

Variable	β	SE	P
**Intercept**	11.07	6.01	<0.0001
**Age**	−0.04	0.10	0.11
**Smoking**			
**Current**	−3.16	1.34	0.003
**Former**	−1.11	0.79	0.11
**BMI**			
**Overweight**	0.47	0.88	0.99
**Obese**	2.51	1.10	0.09
**Time**			
**10AM–12PM**	−4.00	0.85	<0.0001
**1PM–4PM**	−5.44	1.02	<0.0001
**Season**			
**Spring**	−2.15	1.26	0.19
**Summer**	−1.01	0.78	0.68
**Fall**	−0.88	2.04	0.77

BMI = Body Mass Index (kg/m2).

Time = Time of the day during sample collection.

Age modeled as a continuous variable.

Never smoker, normal weight, 7AM–9AM, and winter used as reference groups for smoking, BMI, Time, and season, respectively.

### Estimates of Power

**Figure 3:**
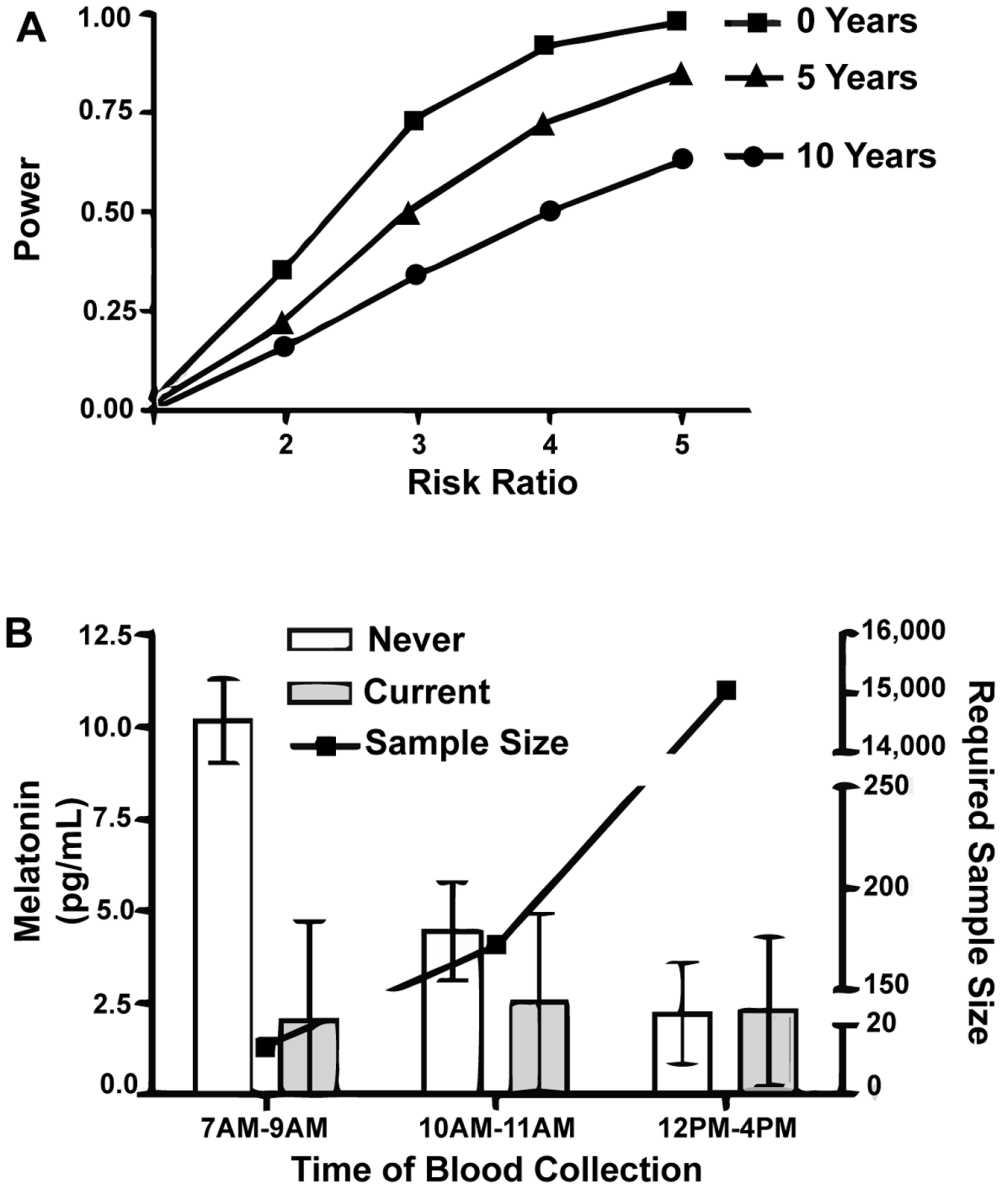
Determinants of study power. **A**. Study power decreases with increasing sample storage time (▪0 years, ▴ 5 years, •10 years). The curves show the corresponding power of a study (n = 50) for each magnitude of relative risk of disease when comparing the highest quartile of melatonin level to the lowest quartile. **B**. Required sample size for detecting differences between subjects is smaller if blood is collected before 9AM (14 men) compared to 10AM–12PM (172 men) and 1PM–4PM (15032 men). Data shown as Mean (Standard Error of the Mean).

Given the low contribution of short term effects to intra-individual melatonin levels variability, measuring melatonin in two samples collected within a short time period (<1 year) offers minimal improvement to the power of a study. However, since serum melatonin levels decrease in samples that have been stored for longer periods, measuring melatonin in samples that have been collected more recently provides more power to identify associations between melatonin levels and outcomes of interest. For example, compared to 50 samples stored for less than one year, 50 samples that have been stored for 5 or 10 years would be equivalent to 23 or 17 samples, respectively. Depending on the risk ratio of the outcome of interest (2, 3, or 4, for example), using samples stored for less than one year would increase the power of the study by 1.56, 1.42, and 1.26 times, respectively (Figure 3A).

The differences in melatonin levels according to lifestyle characteristics and environmental factors were more easily detectable when samples were collected before 9AM (Table S2). In order to evaluate the effect of time of the day during sample collection on study power, we used smoking as example of the variable of interest. The mean (standard deviation) serum melatonin levels in never smokers and current smokers measured between 7AM and 9AM were 10.16 (8.15) pg/ml and 2.03 (2.52) pg/ml. In contrast, the levels measured between 1PM and 4PM were 2.21 (0.99) pg/ml for current smokers and 2.30 (1.81) pg/ml in never smokers. Given these estimates, 14 subjects would be needed to have 80% power to detect the effect of smoking in samples collected before 9AM, while 15,032 subjects would be needed if samples were collected between 1PM and 4PM **(** **Figure 3B** **)**.

## Discussion

In this study of healthy non-Hispanic white males, we showed that 1) correlation of intra-individual serum melatonin levels decreases over a 5 year period, 2) identifying differences in serum melatonin levels between individuals is easier in samples collected before 9AM, and 3) smoking status affect serum melatonin levels in men.

To our knowledge, this is the first study to evaluate long-term intra-person variation in serum melatonin levels and its impact on future studies of disease-melatonin associations. Furthermore, we assessed the role of lifestyle and environmental factors in that variation.

The correlation between serum melatonin levels decreased over time, concordant with urinary aMT6S measurements, where the correlation decreases from r = 0.85 over 72 hours to [69], [84] r = 0.75 in 6 months [51], r = 0.72 in 3 years, [71], and r = 0.56 in 5 years [80]. Factors influencing individual changes in melatonin levels over time include the subject's age and sample storage time. Serum melatonin levels decreased with advancing age at rates similar to that reported in cross-sectional studies [51], [62], [71], [78], [79], and melatonin levels decreased with increasing number of years in storage. Progressive degradation associated with long-term sample storage has only recently been reported [85], and its role on melatonin measurements has not been studied. These results suggest that epidemiologic studies evaluating the role of melatonin in cancer etiology should match on individual's age and sample storage time in order to minimize bias. Additionally, while measuring multiple samples collected at least 5 years apart can increase study power, laboratory variability was minimal, indicating that one measurement is representative of melatonin levels at a single time point.

Similar to studies conducted in Nordic latitudes [51], [63]–[67], where differences in day length between seasons is more pronounced than in tropical latitudes, we found that melatonin levels were slightly higher during the winter. During the winter, melatonin circadian rhythm also displays a phase shift towards the morning hours [63], [64], [66], [67], [86]–[88], with shorter duration of melatonin excretion [79] which could attenuate seasonal differences in melatonin levels if samples are collected in the morning, as they were in our study.

In epidemiologic studies, melatonin is traditionally measured using first-void urinary aMT6s, which reflects melatonin levels during the night [89]. There is good correlation between urine aMT6s and serum melatonin levels [72] and blood samples, not first-void urine, are routinely collected in epidemiology studies. Our data suggest that for studies that measured serum melatonin during the day, it is best to use samples collected in early morning (e.g. before 9AM), when melatonin levels are reasonably high and allow for evaluating differences between individuals, as indicated by our power analysis. Concordant with previous studies which have reported that serum melatonin levels measured in samples collected after 10AM cannot be used as a biomarker for sleep duration [90] or exposure to light at nigh [91], we do not recommend using samples collected after 10AM. Samples collected after 10AM are problematic because the individual variation might be smaller than the assay technical variation.. Finally, epidemiology studies should always consider time of the day during blood collection when evaluating associations with serum melatonin levels.

Smoking status was the only variable to have a significant relative impact on melatonin variability when all environmental and behavioral characteristics were evaluated together with time of the day during sample collection. Our results show that male current smokers had melatonin levels that were 50% those of former and never smokers, similar to the ratio previously reported for healthy adult women [71], [92]. This is the largest study to date to evaluate melatonin levels associated with smoking status in men [93] and these results need to be replicated in larger studies.

In this population of healthy adult men, melatonin levels were slightly higher in obese men, similar to results presented in a recent study [94] and two early small studies [95], [96]. This positive association between melatonin and BMI in males is intriguing, since an inverse association is observed in women [51], [62], [71], [84], [97]. The relationship between melatonin levels and BMI has been shown to be gender specific in pubertal individuals, where boys displayed a positive association between BMI and melatonin levels [98]. Further studies with larger sample of males are required to clarify the association, if any, between melatonin and BMI in men.

Our study has several limitations. Although we included more than 200 samples for the assessment of seasonal variation, our sample size is still limited. Additionally, self-reported BMI is subject to misclassification [99], and the well-known downward bias in self-reported BMI could have attenuated our results. For the melatonin variability over time analysis, BMI and smoking status were only assessed at baseline and might have changed during the five years of follow up. Hence, we were unable to account for potential differences in melatonin levels due to changes in BMI and smoking status through time. Finally, our data are limited to non-Hispanic whites, and patterns in melatonin levels need to be investigated further in other ethnicities.

Strengths of the study should be noted. We were able to identify the contribution of assay reliability, sample storage time, and lifestyle characteristics to intra-individual variation in serum melatonin levels over time. Accuracy in estimating intra-individual variability was increased by collecting samples during the same season and time of day at all time points for each individual. Also, this is the largest study to evaluate differences in serum melatonin levels due to smoking and BMI in healthy males [95], [96] to date. Previous studies have focused mainly in women [14], [51], [62], [84], [97].

In summary, our results provide important insight for accurately measuring melatonin levels in previously collected serum samples. Future study designs should consider our results, as well as the fact that serum melatonin levels reflect the amount of circulating melatonin at the time of sample collection and is not a comprehensive biomarker for the different aspects of the melatonin profile [73], to appropriately address their research question.

## Supporting Information

Table S1 Characteristics of Participants with Samples Collected during the Summer and the Winter.
(DOC)

Table S2 Environmental and Lifestyle Characteristics Comparisons According to Time of the Day during Sample Collection.
(DOC)
