# Supplementary material for: Individual Variations in Serum Melatonin Levels through Time: Implications for Epidemiologic Studies
Source: PLoS One. 2013 Dec 23;8(12):e83208. doi: 10.1371/journal.pone.0083208 (PMC3871612; doi:10.1371/journal.pone.0083208)
Supplement: Table S1 — Characteristics of Participants with Samples Collected during the Summer and the Winter. (DOC) [file pone.0083208.s001.doc]

**Table S1**. **Characteristics of Participants with Samples Collected during the Summer and the Winter**

|  | **Winter (n=130)** | | **Summer (n=119)** | | **P Value** |
| --- | --- | --- | --- | --- | --- |
|  | **Mean** | **SEM** | **Mean** | **SEM** |  |
| **Age (years)** | 63.32 | 0.32 | 63.24 | 0.33 | *0.8* |
| **BMI (kg/m^2^)** | **N** | **%** | **N** | **%** | *0.3* |
| Normal | 37 | 28.46 | 31 | 26.05 |
| Overweight | 65 | 50 | 70 | 58.82 |
| Obese | 28 | 21.54 | 18 | 15.13 |
| **Time of the day** |  |  |  |  | *0.7* |
| 7AM-9AM | 69 | 53.08 | 57 | 47.9 |
| 10A-12PM | 36 | 27.69 | 37 | 31.09 |
| 1PM-4PM | 25 | 19.23 | 25 | 21.01 |
| **Smoking** |  |  |  |  | *0.3* |
| Never | 43 | 33.08 | 38 | 31.93 |
| Current | 10 | 7.69 | 16 | 13.45 |
| Former | 77 | 59.23 | 65 | 54.62 |

SEM = Standard Error of the Mean. BMI = Body Mass Index. There were no differences in age, BMI, time of the day during blood collection, or smoking status between the two groups of individuals
