# Supplementary material for: Individual Variations in Serum Melatonin Levels through Time: Implications for Epidemiologic Studies
Source: PLoS One. 2013 Dec 23;8(12):e83208. doi: 10.1371/journal.pone.0083208 (PMC3871612; doi:10.1371/journal.pone.0083208)
Supplement: Table S2 — Environmental and Lifestyle Characteristics Comparisons According to Time of the Day during Sample Collection. (DOC) [file pone.0083208.s002.doc]

**Table S2. Environmental and Lifestyle Characteristics Comparisons According to Time of the Day during Sample Collection**

| **Time** | **7AM-9AM** | | **10AM-12PM** | | **1PM-4PM** | | **Collapsed** | |
| --- | --- | --- | --- | --- | --- | --- | --- | --- |
| **Melatonin pg/mL** | **Mean (SEM)** | **N** | **Mean (SEM)** | **N** | **Mean (SEM)** | **N** | **Mean (SEM)** | **N** |
| **Season** |  |  |  |  |  |  |  |  |
| **Winter** | 8.67 (0.77) | 69 | 4.79 (1.06) | 36 | 2.23 (1.27) | 25 | 6.36 (0.59) | 130 |
| **Summer** | 7.26 (0.85) | 57 | 2.91 (1.05) | 37 | 2.21 (1.27) | 25 | 4.83 (0.62) | 119 |
| ***P* value** | 0.2 |  | 0.2 |  | 0.9 |  | 0.07 |  |
| **Smoking** |  |  |  |  |  |  |  |  |
| **Never** | 10.16 (1.15) | 45 | 4.45 (1.35) | 21 | 2.21 (1.39) | 15 | 6.67 (0.67) | 81 |
| **Current** | 2.03 (2.70) | 13 | 2.53 (2.41) | 6 | 2.30 (2.04) | 7 | 3.02 (1.26) | 26 |
| **Former** | 7.72 (1.12) | 68 | 4.22 (1.00) | 46 | 2.21 (1.02) | 28 | 5.60 (0.50) | 142 |
| ***P* value** | **<0.01** |  | 0.4 |  | 0.9 |  | **0.01** |  |
| **BMI** |  |  |  |  |  |  |  |  |
| **Normal** | 6.71 (0.96) | 36 | 3.59 (1.47) | 16 | 2.81 (1.52) | 16 | 5.13 (0.76) | 68 |
| **Overweight** | 7.01 (0.67) | 63 | 4.08 (0.86) | 45 | 1.95 (1.17) | 27 | 5.24 (0.52) | 135 |
| **Obese** | 11.21 (1.04) | 27 | 3.04 (1.52) | 12 | 1.92 (2.30) | 7 | 7.77 (0.86) | 46 |
| ***P* value** | 0.06 |  | 0.3 |  | 0.7 |  | 0.06 |  |

SEM = Standard Error of the Mean. BMI = Body Mass Index (kg/m^2^). Normal between 18 and 25 kg/m^2^, overweight between 26 and 30 kg/m^2^, and obese above 30 kg/m^2^. *P* < 0.05 for comparison between never and current smokers between 7AM-9AM and all times combined.
